# The important role of biochemical and functional studies in the diagnostics of peroxisomal disorders

**DOI:** 10.1007/s10545-016-9922-4

**Published:** 2016-03-04

**Authors:** Sacha Ferdinandusse, Merel S. Ebberink, Frédéric M. Vaz, Hans R. Waterham, Ronald J. A. Wanders

**Affiliations:** Laboratory Genetic Metabolic Diseases, Academic Medical Center, University of Amsterdam, Meibergdreef 9, 1105 AZ Amsterdam, The Netherlands

## Abstract

Peroxisomes are dynamic organelles that play an essential role in a variety of metabolic pathways. Peroxisomal dysfunction can lead to various biochemical abnormalities and result in abnormal metabolite levels, such as increased very long-chain fatty acid or reduced plasmalogen levels. The metabolite abnormalities in peroxisomal disorders are used in the diagnostics of these disorders. In this paper we discuss in detail the different diagnostic tests available for peroxisomal disorders and focus specifically on the important role of biochemical and functional studies in cultured skin fibroblasts in reaching the right diagnosis. Several examples are shown to underline the power of such studies.

## Peroxisomal disorders

Peroxisomal disorders form a group of inherited disorders caused by mutations in genes encoding peroxisomal proteins, which lead to one or more impaired peroxisomal functions or cause the absence of functional peroxisomes altogether. Peroxisomes are dynamic organelles that play an essential role in a variety of cellular catabolic and anabolic metabolic pathways, including fatty acid alpha- and beta-oxidation, and plasmalogen and bile acid biosynthesis (Wanders and Waterham 2006a). A defect of one or more functions of the peroxisome leads to abnormal metabolite levels, which is used in the screening for peroxisomal disorders (Wanders and Waterham 2006a, b; Van Veldhoven 2010). Peroxisomal disorders can be divided in two main subgroups: 1) the peroxisome biogenesis disorders (Steinberg et al 2006; Waterham and Ebberink 2012) and 2) the single enzyme defects (Wanders and Waterham 2006b; Van Veldhoven 2010). Both the clinical and genetic spectrum of the peroxisomal disorders is still expanding. The peroxisome biogenesis disorders include not only the Zellweger spectrum disorders (ZSDs, with an estimated incidence of 1 in 50,000 newborns in the United States) and Rhizomelic chondrodysplasia punctata (RCDP) type 1 (OMIM 215100), but also the recently identified RCDP type 5 (Baroy et al 2015) and a novel subclass of peroxisomal fission disorders (i.e. PEX11β (OMIM 614920) (Ebberink et al 2012; Thoms and Gartner 2012), DLP/DRP1 (OMIM 614388) (Waterham et al 2007) and MFF (Shamseldin et al 2012) deficiency). The group of the single enzyme defects has expanded in the last 2 years with two disorders, i.e. RCDP type 4 (FAR1 deficiency, OMIM 616154) (Buchert et al 2014) and ABCD3 deficiency (OMIM 616278) (Ferdinandusse et al 2015). The clinical spectrum is expanding mainly with the recognition of very mild forms of peroxisomal disorders. For example, Heimler syndrome was recently identified as a very mild form of a peroxisomal biogenesis disorder (Ratbi et al 2015), and D-bifunctional protein (DBP) deficiency (OMIM 261515) has been shown to be one of the possible causes in patients with Perrault syndrome (Pierce et al 2010). The different peroxisomal disorders known to date and the peroxisomal metabolic pathways that are affected in these disorders are summarized in Table 1, but will not be discussed in detail in this paper, which focuses primarily on the diagnostics of peroxisomal disorders.

**Table 1:** Peroxisomal disorders and the affected metabolic pathways in these disorders

Peroxisomal disorder	Affected metabolic pathway
Peroxisome biogenesis disorders	
Zellweger spectrum disorders (ZSDs)	Phytanic acid alpha-oxidation
	VLCFA and pristanic acid beta-oxidation
	Plasmalogen biosynthesis
	Bile acid biosynthesis
	Docosahaxaenoic acid (DHA) biosynthesis
Rhizomelic chondrodysplasia punctata (RCDP) type 1 and 5	Plasmalogen biosynthesis
	Phytanic acid alpha-oxidation
Peroxisomal fission disorders	No obvious biochemical abnormalities
Single enzyme defects	
Rhizomelic chondrodysplasia punctata (RCDP) type 2-4	Plasmalogen biosynthesis
Refsum disease	Phytanic acid alpha-oxidation
Alpha-methylacyl-CoA racemase (AMACR) deficiency	Pristanic acid beta-oxidation
	Bile acid biosynthesis
Sterol carrier protein X (SCPx) deficiency	Pristanic acid beta-oxidation
	Bile acid biosynthesis
ABCD3 deficiency	Bile acid biosynthesis
Acyl-CoA oxidase 1 (ACOX1) deficiency	VLCFA beta-oxidation
X-linked adrenoleukodystrophy (X-ALD)	VLCFA beta-oxidation
D-bifunctional protein (DBP) deficiency	VLCFA beta-oxidation
	Pristanic acid beta-oxidation
	Bile acid biosynthesis
	Docosahaxaenoic acid (DHA) biosynthesis
Bile acid-CoA:amino acid N-acyltransferase (BAAT) deficiency	Bile acid conjugation
Primary hyperoxaluria	Glyoxylate detoxification
Acatalasemia	Hydrogen peroxide detoxification

**Fig. 1:**
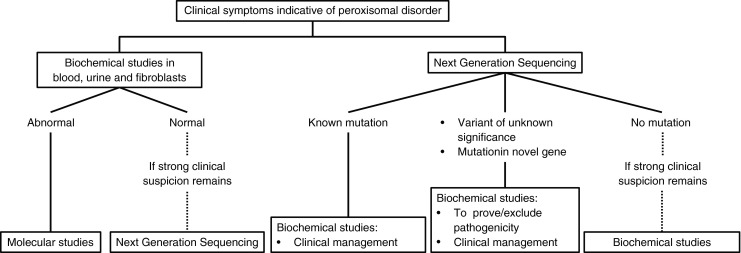
Diagnostic flow-chart for peroxisomal disorders

Ever since their first identification, the laboratory diagnosis of the peroxisomal disorders has relied on biochemical measurements in patient materials and, at this moment, this is still the main diagnostic route. If a peroxisomal disorder is suspected based on the clinical presentation, peroxisomal metabolites are measured in blood and urine. When abnormal levels of these metabolites are found, further studies are performed in cultured fibroblasts, which will either exclude a peroxisomal disorder or will provide a diagnosis. Subsequent molecular analysis will allow identification of the underlying mutations. Now that next generation sequencing (NGS) is becoming more readily available, an increasing number of patients is being identified via NGS. However, also when NGS is used in diagnostics, biochemical and functional studies in fibroblasts often remain essential, firstly to confirm that the correct diagnosis has been made, secondly to prove pathogenicity of previously uncharacterized mutations and thirdly to study the severity of the defect which is necessary for proper clinical management of the patient. Figure 1 shows a diagnostic flow-chart for peroxisomal disorders. In this paper, a detailed review of peroxisomal diagnostics is provided, as performed in the Laboratory Genetic Metabolic Diseases at the Academic Medical Center in Amsterdam since the early 1980s, illustrated with examples demonstrating potential pitfalls.

## Screening for peroxisomal disorders in blood and urine

To confirm a clinical suspicion of a peroxisomal disorder by laboratory testing, peroxisomal metabolites should be measured in blood and urine. These metabolites include: 1) Very long-chain fatty acids (VLCFAs) in plasma 2) C26:0 lyso PC and C26-acylcarnitine in blood spot 3) phytanic acid and pristanic acid in plasma, 4) THCA and DHCA in plasma and urine (as part of bile acid analysis), 5) pipecolic acid in plasma and 6) plasmalogens in erythrocytes.

*VLCFAs* are straight-chain fatty acids with a chain length of 22 carbon atoms or more. VLCFAs are endogenously synthesized via chain elongation of long-chain fatty acids and accumulate in case peroxisomal beta-oxidation is impaired. VLCFAs analysis in plasma using gas-chromatography mass spectrometry is the most widely used laboratory test for peroxisomal disorders (Vreken et al 1998). In this test, all lipids are hydrolyzed during sample work-up so that total levels of C26:0, C24:0 and C22:0 are measured. This allows calculation of the C26/C22 ratio, which is increased in ZSD, X-ALD (OMIM 300100), ACOX1 (OMIM 264470) and DBP deficiency. Recently, C26:0 lyso PC measurement in blood spot using liquid chromatography tandem mass spectrometry (LC-MS/MS) has been introduced as a screening parameter (Theda et al 2014) but this is operational in a limited number of laboratories only. C26:0 lyso PC is a marker for C26:0 accumulation in the phospholipid fraction. The sample work-up is simple and the method is already used for newborn screening for X-ALD in the state of New York and will be introduced in other countries in the near future. As another biomarker for VLCFA accumulation, C26-carnitine can be measured in blood spot using LC-MS/MS (Rizzo et al 2003).

It is important to mention that VLCFAs may be abnormal in the absence of peroxisomal dysfunctions. For example, excess dietary intake via peanut consumption (Lam et al 2012) or a ketogenic diet (Theda et al 1993) can lead to increased levels of plasma VLCFAs, which is a diagnostic pitfall.

*Phytanic and pristanic acid* measurement can be incorporated in the VLCFA analysis when an alkaline hydrolysis step is included in the work up of the sample (Vreken et al 1998). The branched-chain fatty acids phytanic and pristanic acid cannot be endogenously synthesized and are exclusively derived via the diet either as phytanic and pristanic acid itself or through their precursor phytol (Wanders et al 2011). Phytol is part of the chlorophyll molecule and can be released by bacteria in the gut of ruminants. Depending on the age and the diet of the patient, the branched-chain fatty acids are increased in Refsum disease (OMIM 266500), RCDP type 1 and 5, ZSDs, DBP, alpha-methylacyl-CoA racemase (AMACR, OMIM 614307), sterol carrier protein X (SCPx, OMIM 613724) and possibly ATP-binding cassette sub-family D member 3 (ABCD3) deficiency. In Refsum disease and RCDP type 1 and 5, the major accumulating metabolite is phytanic acid due to a defect of alpha-oxidation, whereas in DBP, AMACR and SCPx deficiency the pristanic/phytanic acid ratio is increased because peroxisomal beta-oxidation of pristanic acid is impaired and phytanic acid accumulation is only secondary. The only patient with ABCD3 deficiency identified so far did not show accumulation of phytanic or pristanic acid but studies in the mouse model did point to a role for ABCD3 in the transport of branched-chain fatty acids into the peroxisome (Ferdinandusse et al 2015). Other, longer surviving ABCD3 patients could therefore possibly be identified with accumulation of the branched-chain fatty acids.

*Bile acid* analysis by electrospray ionization-tandem mass spectrometry (ESI-MS/MS) in plasma and urine provides information about the primary C24-bile acids cholic acid and chenodeoxycholic acid but also about the peroxisomal C27-bile acid intermediates di- and trihydroxycholestanoic acid (D/THCA) and the C29-dicarboxylic acid (Bootsma et al 1999). Bile acids are formed in the liver from cholesterol and, in the major pathway for bile acid biosynthesis, peroxisomal shortening of the side-chain by one cycle of beta-oxidation is required (Ferdinandusse and Houten 2006). C27-bile acids can be detected in plasma and urine from patients with a ZSD as well as in DBP, AMACR, SCPx and ABCD3 deficiency (Ferdinandusse and Houten 2006; Ferdinandusse et al 2009, 2015; Clayton 2011). C29-dicarboxylic acid, which is formed via chain-elongation of THC-CoA, is only found in plasma because it is poorly excreted in urine. Characteristic for AMACR deficiency is the exclusive accumulation of (25*R*)-DHCA and (25*R*)-THCA since they require racemization by AMACR before peroxisomal beta-oxidation of the side-chain can occur. In DBP deficiency, another important accumulating C27-bile acid intermediate is 24-OH-THCA. In SCPx deficiency, only trace amounts of DHCA and THCA are present but urine bile acid analysis reveals a characteristic excretion of bile alcohol glucuronides (m/z 611, 613, 627, 629, 643 and 645). In urine, hydroxylated forms of THCA are present since hydroxylation increases polarity and thereby urinary excretion. The presence of the taurine conjugate of hydroxylated 24-ene-THCA (m/z 570) and dihydroxylated 24-ene-THCA (m/z 586) is characteristic for patients with DBP deficiency.

Both in ZSDs and DBP deficiency it has been shown that the extent of the peroxisome/DBP deficiency corresponds to the extent of the deficiency of bile acid biosynthesis (Gootjes et al 2002; Ferdinandusse et al 2006a, b; Berendse et al 2015). Patients with a milder clinical presentation and longer survival tend to accumulate less DHCA and THCA. C27-bile acids can even be completely normal; in a large cohort of DBP-deficient patients it was shown that in 26 % of the patients no C27-bile acids were present in plasma (Ferdinandusse et al 2006a, b).

*Pipecolic acid* analysis by ESI-MS/MS in plasma (or urine) is a useful parameter in the diagnosis and classification of peroxisomal disorders (Peduto et al 2004). Since L-pipecolic acid oxidase, involved in the breakdown of pipecolic acid, is a peroxisomal enzyme, pipecolic acid accumulates in ZSDs but not in peroxisomal single enzyme defects.

Measurement of *plasmalogens* by gas chromatography (GC) in erythrocytes should be included in the screening for peroxisomal disorders to investigate a possible defect in plasmalogen biosynthesis (Dacremont and Vincent 1995). Plasmalogens are etherphospholipids and the first two steps of the plasmalogen biosynthesis pathway are peroxisomal (Brites et al 2004; Braverman and Moser 2012). The main biochemical defect in all types of RCDP is a deficiency of plasmalogens but also in the ZSDs, there is a plasmalogen deficiency. The phenotype of patients within the RCDP spectrum correlates well with plasmalogen levels, with the lowest levels of plasmalogens in erythrocytes in patients with the severe phenotype (Braverman et al 2002; Bams-Mengerink et al 2013). In ZSDs, plasmalogen levels are low in severely affected patients and can be completely normal in milder patients.

## Peroxisomal studies in cultured skin fibroblasts

Studies of peroxisomes and peroxisomal functions in cultured primary skin fibroblasts are important and even can be essential in the diagnostic process for peroxisomal disorders. Several tests are available to study different aspects of peroxisomes and their functions (see Table 2).

**Table 2:** Functional tests available for peroxisomal diagnostics in cultured skin fibroblasts

*Metabolite measurement*
Very long-chain fatty acid analysis
C26:0 lyso phosphatidylcholine (C26:0 lyso PC)
Plasmalogens
*Immuno(cyto/bio)chemical assays*
Immunoblotting peroxisomal proteins
Immunofluorescence microscopy analyses
*Enzyme activity measurements*
Dihydroxyacetonephosphate-acyltransferase (DHAPAT)
Acyl-CoA oxidase 1 (ACOX1)
D-bifunctional protein (DBP)
Sterol carrier protein X (SCPx)
*Pathway analyses*
Phytanic acid alpha-oxidation activity with [1-^14^C] phytanic acid
Pristanic acid beta-oxidation activity with [1-^14^C] pristanic acid
C26:0 beta-oxidation with [1-^14^C] C26:0
D3-C22 loading test (VLCFA metabolism)

*First*, as in blood, specific peroxisomal metabolites can be measured in cultured skin fibroblasts. These include VLCFAs, C26:0 lyso PC and plasmalogens (Dacremont et al 1995; Dacremont and Vincent 1995). VLCFAs and C26:0 lyso PC are abnormal in ZSDs, X-ALD, ACOX1 and DBP deficiency, and plasmalogens are deficient in ZSDs and RCDP.

**Fig. 2:**
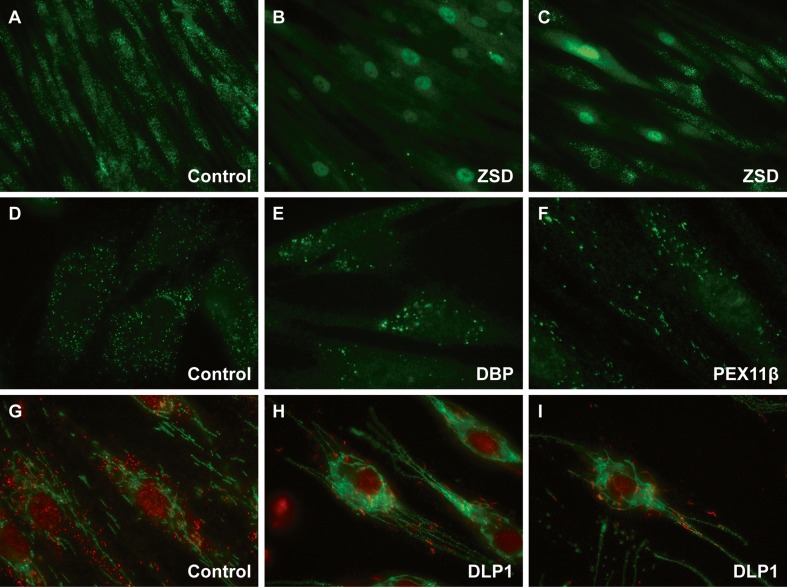
Microscopical analyses of cultured skin fibroblasts. Immunofluorescence microscopy analyses using a specific antiserum against catalase, a peroxisomal matrix enzyme, reveals a typical punctuated staining in fibroblasts of a control subject (**a** and **d**). In severe Zellweger spectrum disorders (ZSD) there is no peroxisomal staining (**b**), but in milder ZSD patients a mosaic pattern can be observed composed of cells with a normal peroxisomal staining, cells with a reduced number of peroxisomes and cells with no peroxisomal staining (**c**). Typically, fibroblasts of patients with D-bifunctional protein (DBP) deficiency (**e**) or acyl-CoA oxidase 1 (ACOX1) deficiency show a reduced number of enlarged peroxisomes. PEX11β deficiency results in elongated and enlarged peroxisomes (**f**). Fibroblasts from DLP1/DRP1 patients (**h** and **i**) have fewer peroxisomes (*red dye*) which are often arranged like beads on a string, and the mitochondria are elongated and interconnected (*green dye*, TOM20 antibody)

*Second*, immunofluorescence microscopy analyses can be performed with antibodies specific for peroxisomal matrix or membrane proteins to study the presence/absence of import-competent peroxisomes and peroxisomal membranes (Wanders et al 1989) (Fig. 2). Catalase immunofluorescence microscopy analysis has proven to be the most sensitive test in fibroblasts for the diagnosis of ZSDs. In mild patients, all biochemical testing in blood and fibroblasts can even be normal, with only an aberrant peroxisomal staining upon immunofluorescence microscopy analysis. In severe patients, import-competent peroxisomes are absent in all cells, but the cells may still contain peroxisomal membrane remnants (ghosts). These ghosts are enlarged when compared to the size of functional peroxisomes. Whether ghosts are still present depends on the function of the defective *PEX* gene but also on the severity of the mutations. In milder patients often a mosaic pattern is observed with cells without peroxisomal staining, cells with a reduced number of peroxisomes and cells with a normal peroxisomal staining. The mosaic pattern varies for all patients, for example the mosaic can exist mainly of cells lacking peroxisomes but also mainly of cells with functional peroxisomes, and the observed pattern also depends on culture conditions. When fibroblasts with a peroxisomal mosaic pattern are cultured at 40 °C, the peroxisome biogenesis defect is often exacerbated resulting in the loss of import-competent peroxisomes (Gootjes et al 2004). Peroxisomes can also be enlarged in ZSDs, although this has only been observed in a minority of patient cells (e.g. PEX16 (Ebberink et al 2010) and PEX11β defects (Ebberink et al 2012; Thoms and Gartner 2012)). Most often, however, enlarged peroxisomes point to a peroxisomal fatty acid oxidation defect. Fibroblasts of ACOX1-deficient patients show a markedly reduced number of very enlarged peroxisomes (Ferdinandusse et al 2007). This phenotype, although less pronounced, can also be observed in fibroblasts of DBP-deficient patients, but not in all cases especially not in fibroblasts of milder patients (Ferdinandusse et al 2006a, b). Fibroblasts of other peroxisomal fatty acid oxidation disorders (i.e. X-ALD, Refsum disease, AMACR and SCPx deficiency) have a normal number of morphologically normal peroxisomes. Next to providing information about the localization of peroxisomal proteins, immunofluorescence microscopy analysis is also useful to study the presence or absence of the peroxisomal proteins for which mutations have been found in the encoding genes. For example, it has been reported that 77 % of all non-recurrent *ABCD1* mutations result in the absence of adrenoleukodystrophy protein (ALDP) (http://www.x-ald.nl) (Kemp et al 2012). In fibroblasts of female ABCD1 carriers, a mosaic pattern can be observed due to X-chromosome inactivation (Kemp et al 2012). In patients with CCADS (contiguous ABCD1 DXS1357E (BAP31) deletion syndrome, OMIM 300475) there is also no expression of ALDP (Corzo et al 2002). The only ABCD3-deficient patient was initially diagnosed by the serendipitous finding of absence of ABCD3 staining upon routine immunofluorescence microscopy analysis when screening for peroxisomal disorders in fibroblasts was performed (Ferdinandusse et al 2015).

**Fig. 3:**
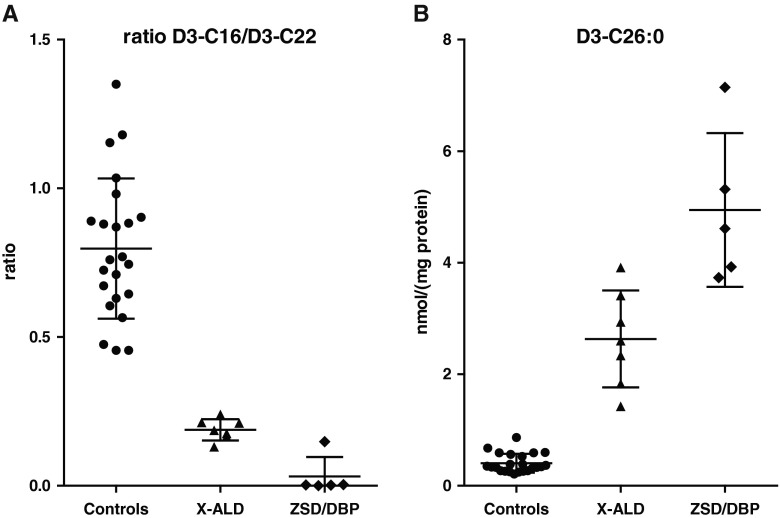
Loading test with D3-C22:0 in cultured skin fibroblasts. Fibroblasts of control subjects and patients with X-linked adrenoleukodystrophy (X-ALD), a Zellweger spectrum disorder (ZSD) and D-bifunctional protein deficiency (DBP) were incubated for 3 days with 30 μM D3-C22:0 followed by fatty acid analysis using tandem mass spectrometry. In the patient cell lines, breakdown of D3-C22:0 is reduced as reflected by a reduced D3-C16:0/D3-C22:0 ratio (**a**) and chain elongation is increased as reflected by an increased D3-C26:0 formation (**b**). Whiskers indicate mean ± standard deviation

*Third*, the flux through the peroxisomal fatty acid alpha- and beta-oxidation systems can be determined with radiolabelled substrates (i.e. [1-^14^C]-phytanic acid, [1-^14^C]-pristanic acid, [1-^14^C]-C26:0) (Wanders and Van Roermund 1993; Wanders et al 1995b). With these methods the end-products of fatty acid oxidation are measured (i.e. [^14^C]CO_2_ and [^14^C]-labelled acid soluble products such as acetate, propionate and Krebs cycle intermediates). Measurement of fatty acid oxidation activities with different substrates allows differentiation between different peroxisomal disorders and therefore provides an effective tool to establish the diagnosis in a patient. In addition, it provides information about the severity of the defect. A correlation between the severity of the clinical presentation and the residual peroxisomal fatty acid oxidation activity has been shown for ZSDs and DBP deficiency (Gootjes et al 2002; Ferdinandusse et al 2006b). Phytanic acid oxidation activity is deficient in fibroblasts of patients with Refsum disease, RCDP type 1 and 5 and ZSDs, but can also be reduced in fibroblasts of DBP-deficient patients most likely due to the reduced number of functional peroxisomes in severe patients. Pristanic acid beta-oxidation activity is deficient in ZSDs and DBP deficiency and only partly deficient in AMACR, SCPx and ABCD3 deficiency. C26:0 beta-oxidation activity is deficient in ZSDs, X-ALD, ACOX1 and DBP deficiency. In addition to these tests with radiolabelled substrates, peroxisomal fatty acid oxidation of straight-chain fatty acids can be studied by loading fibroblasts for 3 days with deuterated (D3) C22:0 followed by fatty acid analysis with tandem mass spectrometry (Kemp et al 2004; Ebberink et al 2012). Labelled and unlabelled fatty acids with chain lengths ranging from 16 carbon atoms up to 30 carbon atoms are measured. Although this test does not provide information about the flux through the beta-oxidation system, since the end products of beta-oxidation are not measured, the test provides information about VLCFA metabolism and allows detection of peroxisomal disorders with a defect in peroxisomal beta-oxidation of VLCFAs (ZSD, X-ALD, ACOX1 and DBP deficiency). Not only a reduced production of D3-C16:0 is observed in these disorders as reflected in a reduced D3-C16/D3-C22 ratio, but also an increased production of D3-C26:0 levels via chain elongation (see Fig. 3).

*Fourth*, enzyme activity measurements can be performed to determine the activity of single enzymes. These include activity measurement of dihydroxyacetonephosphate acyltransferase (DHAPAT), ACOX1, DBP and SCPx. DHAPAT activity is deficient in RCDP type 2 (OMIM 222765) caused by mutations in the encoding gene *GNPAT*, but is also reduced in all other types of RCDP, except RCDP type 4 (FAR1 deficiency), and in ZSDs (Brites et al 2004; Braverman and Moser 2012). This is caused by impaired import of DHAPAT into the peroxisome and/or impaired import of alkyl-DHAP-synthase (AGPS) in combination with the fact that DHAPAT is only stable and can only function optimally when it forms an intra-peroxisomal complex with functional AGPS (de Vet et al 1999; Itzkovitz et al 2012). It should be noted that in attenuated forms of RCDP type 1 and 5, and in RCDP type 3 (OMIM 600121) with normal, but non- functional, protein levels of AGPS, DHAPAT activity can be (near) normal.

ACOX1 activity can be measured in fibroblast homogenates with C16-CoA as substrate in the presence of FAD, followed by separation and detection of the products C16:1-CoA and 3-OH-C16-CoA using (ultra-)high pressure liquid chromatography ((U)HPLC) on a reversed-phase column. ACOX1 activity is deficient in patients with mutations in the encoding gene (Ferdinandusse et al 2007). To some extent there is a correlation between clinical severity and residual activity, but it should be noted that the other peroxisomal oxidase (ACOX2, branched-chain specific) also displays some activity towards C16-CoA as substrate (Vanhove et al 1993), hampering an accurate determination of residual ACOX1 activity. ACOX1 activity is normal in fibroblasts of ZSD patients because activity measurement is performed in a cell homogenate and ACOX1 is stable in the cytosol, at least in fibroblasts. ACOX1 activity is increased in DBP deficiency due to upregulation of its expression (Ferdinandusse et al 2003).

DBP and SCPx can be measured in a single assay using THC:1-CoA as substrate, followed by separation and detection of the products using (U)HPLC on a reversed-phase column (van Grunsven et al 1999; Ferdinandusse et al 2006a, b). In this assay, the substrate for the SCPx reaction is formed by endogenous DBP activity, which could in theory be a problem when SCPx diagnostics is requested in a DBP-deficient patient. In practice, however, this will not occur since clinically and biochemically these disorders are very different. In all DBP-deficient patients, DBP activity is markedly reduced. Remarkably, this is also the case in mild patients with normal peroxisomal parameters in blood and without VLCFA accumulation in fibroblasts (Ferdinandusse et al 2006a; McMillan et al 2012; Lines et al 2014). DBP activity measurement can also be performed in lymphocytes, allowing rapid screening for DBP deficiency in blood. DBP activity in fibroblast homogenates from ZSD patients is somewhat reduced but not fully deficient, indicating that DBP can be active in the cytosol.

**Fig. 4:**
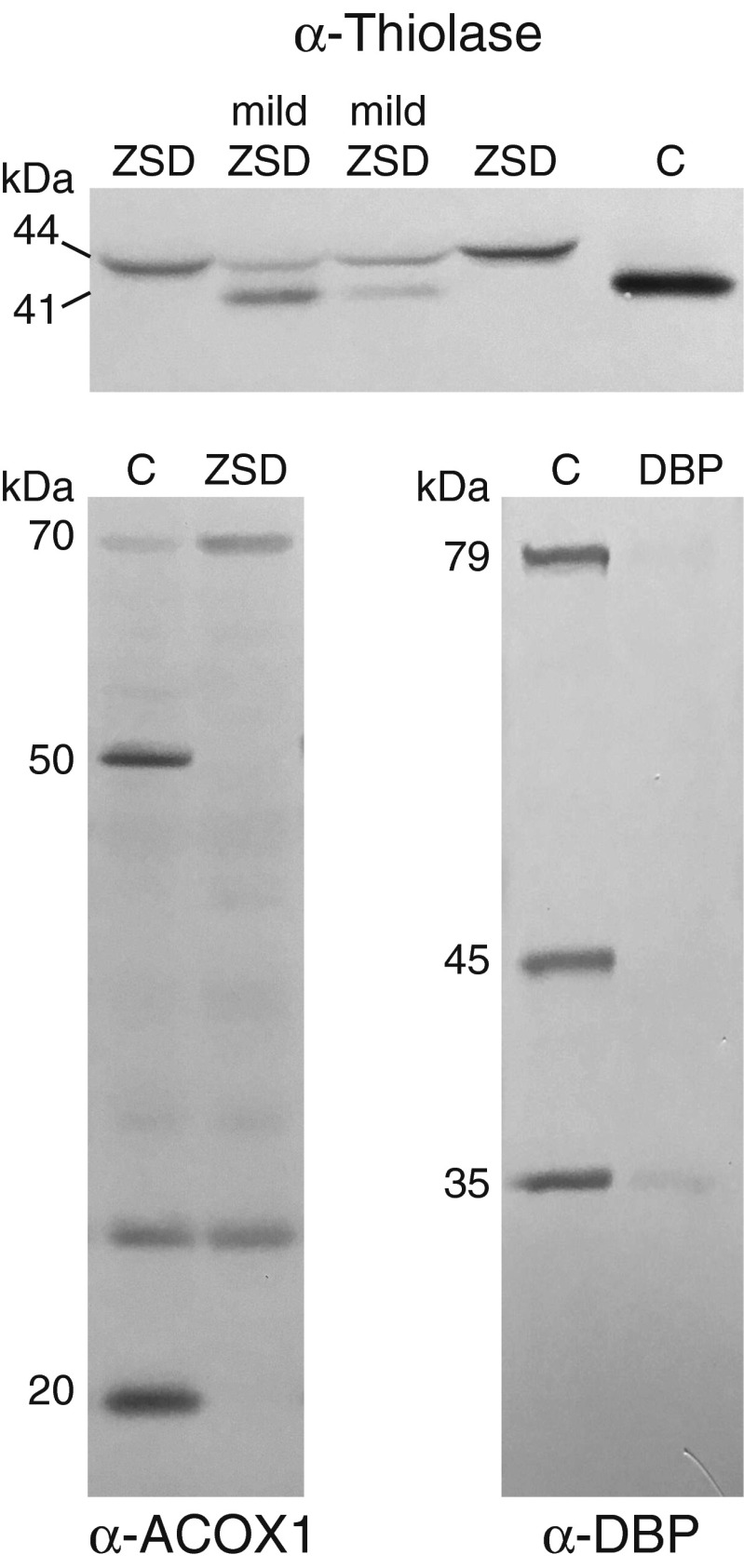
Immunoblot analysis in fibroblast homogenates. Immunoblot analysis with antibodies against peroxisomal 3-ketoacyl-CoA thiolase reveals the mature processed form of 41 kDa in control fibroblasts (C), but the unprocessed form of 44 kDa in fibroblasts of a patient with a Zellweger spectrum disorder (ZSD). In mild ZSDs both forms can be present. Immunoblot analysis with antibodies against acyl-CoA oxidase 1 (ACOX1) reveals the full length protein of 70 kDa and the processed forms of 50 and 20 kDa in control fibroblasts, but in ZSD fibroblasts only the 70 kDa is observed. Immunoblot analysis with antibodies against D-bifunctional protein (DBP) reveals the absence of protein in a DBP-deficient patient

*Fifth*, immunoblot analysis can be performed to study the expression of peroxisomal proteins but also the processing of peroxisomal proteins (Wanders et al 1995a). Several peroxisomal proteins are processed inside the peroxisomes by a protease and thus the immunoblot profiles of these proteins provide information about the presence of import-competent peroxisomes in fibroblasts of a patient and the severity of the potential peroxisome biogenesis defect. ACOX1 and peroxisomal 3-ketoacyl-CoA thiolase immunoblotting are often performed in peroxisomal diagnostics for this purpose (Fig. 4). Especially processing of 3-ketoacyl-CoA thiolase provides valuable information not only about ZSDs but also allows distinction between the different types of RCDP. Processing of 3-ketoacyl-CoA thiolase does not occur in RCDP type 1 and the recently reported type 5. In mild ZSDs, both the unprocessed and processed form of peroxisomal 3-ketoacyl-CoA thiolase can be observed (see Fig. 4).

Immunoblot analysis is an important test in biochemical prenatal testing for ZSDs in chorionic villous biopsy material but also for DBP deficiency as an additional test next to enzyme activity measurement in case the index patient has proven to have no DBP protein expression (Fig. 4).

*Sixth*, complementation studies can be performed in fibroblasts without import-competent peroxisomes to determine which *PEX* gene is defective in the patient’s cells and subsequently allowing direct sequencing of this gene. To this end, a PEX cDNA transfection assay has been developed in which the different pcDNA3-PEX expression vectors are co-transfected separately but together with eGFP-SKL into patient fibroblasts (Ebberink et al 2011). If the correct PEX cDNA is transfected complementation occurs and restoration of peroxisome biogenesis can be observed by fluorescence microscopy. This assay can also be performed at 40 °C for cell lines which display different degrees of peroxisomal mosaicism that are sensitive to culturing at 40 °C, which exacerbates the defect in peroxisome biogenesis. In addition, this complementation assay can be used to show that defects identified by NGS are responsible for the phenotype observed in patient cells. For example, genetic complementation was used to demonstrate that variants in the *PEX1* and *PEX6* genes identified with whole exome sequencing are the cause of the aberrant peroxisomal phenotype in fibroblasts of Heimler syndrome patients (Ratbi et al 2015).

## The importance of functional studies in fibroblasts for peroxisomal diagnostics

Since the discovery in 1973 that Zellweger syndrome is caused by the absence of functional peroxisomes (Goldfischer et al 1973), studies have been performed in fibroblasts of peroxisomal patients to investigate the role of peroxisomes in metabolism. These studies have led to more insight in the different functions of peroxisomes and the metabolic pathways localized partly or fully in peroxisomes, and to the development of many different tests which can be used for peroxisomal diagnostics. When diagnostics for peroxisomal disorders is performed based on clinical presentation and metabolite abnormalities in blood, follow-up studies in fibroblasts are essential to establish the correct diagnosis. Also when NGS techniques are used, functional studies in fibroblasts remain an important part of the diagnostic process. Below, the value of fibroblast studies in peroxisomal diagnostics will be illustrated with different examples.

Based on the clinical presentation and metabolite abnormalities in blood solely, it can be hard or even impossible to distinguish between different peroxisomal disorders.

At the severe end of the clinical spectrum, in the neonatal-infantile and childhood period, the clinical presentation of DBP and ACOX1 deficiency can be indistinguishable from ZSDs, with severe hypotonia, seizures, liver disease and dysmorphia (Ferdinandusse et al 2006a, 2007; Poll-The and Gartner 2012). Also MRI-features in DBP deficiency resemble those of ZSDs (van der Knaap et al 2012). VLCFAs are abnormal in all of these disorders and in the infantile period, dietary-dependent phytanic acid and pristanic acid levels are not yet elevated in ZSDs and DBP deficiency and thus cannot be used for discrimination between the different disorders. Bile acid and plasmalogen analysis would allow differentiation between these defects in severely affected patients, but this is only performed in a limited number of laboratories. In these cases, fibroblast studies should be performed to establish the diagnosis in the patient (see Table 3 for comparison of fibroblasts studies in a classical ZSD and a DBP patient).

**Table 3:** Results of peroxisomal investigations in blood and fibroblasts of different patients diagnosed with a peroxisomal disorder

Patient^a^		1	2	3	4	5^b^	6	7
Diagnosis	Reference range*	Severe ZSD	Mild ZSD (childhood presentation)	Mild ZSD (adult presentation)	Severe DBP	Mild DBP (adult presentation)	RCDP type 1 (Refsum like phenotype)	X-ALD
Blood								
C26:0 (*μmol/L)*	0.45–1.3	**19**		1.0		0.55		**2.7**
C26/C22	0–0.02	**1**		0.02		0.004		**0.04**
C24/C22	0.57–0.92	**2.6**				0.30		**1.1**
Phytanic acid (*μmol/L)*	0.49–9.9	3.4		**39**		1.6	**700**	
Pristanic acid (*μmol/L)*	0.12–3.0	0.90		**51**				
Pipecolic acid (*μmol/L)*	0.1–7	**216**						
DHCA (*μmol/L)*	0–0.02	**4**						
THCA (*μmol/L)*	0–0.08	**18**						
C29DCA (*μmol/L)*	0–0.001	**2**						
C16DMA *(%)*	5.5–8.1	**1.5**						
C18DMA *(%)*	11.8–19.4	**5.7**						
Fibroblasts								
DHAPAT *(nmol/(2 h.mg))*	5.9–16	**0.8**	14	6.0			**2.3**	
C26:0 lyso PC	2–14		8					
C26:0 *(μmol/g)*	0.16–0.41	**1.6**	0.26	**0.64**		0.3		**1.0/0.79/1.1**
C26/C22	0.030–0.10	**0.32**	**0.11**	**0.25**		2.4		**0.17/0.28/0.24**
DBP hydratase *(pmol/(min.mg))*	115–600			120	**26**	**33**		
DBP dehydrogenase *(pmol/(min.mg))*	25–300			41	**<12**	**<12**		
SCPx *(pmol/(min.mg))*	5–68			13				
α-oxidation *(pmol/(min.mg))*	28–95	**2**			**39**	70	**6**	
C26:0 β-oxidation *(pmol/(min.mg))*	800–2040	**193**			**157**	1096		
Pristanic acid β-oxidation *(pmol/(min.mg))*	790–1690	**13**			**<37**	**427**		
Immunoblot profile		ACOX1 AbN	ACOX1 N	–	–	DBP AbN	ACOX1 N	–
		Thio AbN	Thio N				Thio AbN	
IF		Absence of import-competent peroxisomes, ghosts present	Mosaic pattern at 37 °C, negative at 40 °C	Mosaic pattern at 37 °C, negative at 40 °C	Reduced number of enlarged peroxisomes	Normal peroxisomal staining	Normal peroxisomal staining	ALDP absent in most but not all cells
Gene		*PEX1*	*PEX13*	*PEX6*	*-*	*HSD17B4*	*PEX7*	*ABCD1*
Mutations		c.2528G > A (p.Gly843Asp)	c.844G > T (p.Asp282Tyr)	c.1802G > A (p.Arg601Gln) c.2435G > A (p.Arg812Gln)	No mutation identified by DNA analysis of HSD17B4	c.1537C > A (p.Pro513Thr	c.225G > C (p.Trp75Cys)	c.1-22C > T
		c.569C > A (p.Ser190*)				c.1628G > C (p.Arg543Pro		

*AbN* abnormal, *N* normal, *ACOX1* acyl-CoA oxidase 1, *Thio* 3-ketoacyl-CoA thiolase, *DBP* D-bifunctional protein, *ZSD* Zellweger spectrum disorder, *X-ALD* X-linked adrenoleukodystrophy, *RCDP* rhizomelic chondrodysplasia punctata, *IF* immunofluorescence microscopy analysis

Values outside reference range have been indicated in bold.

^a^Patients are described in the main text, the patient numbers correspond to the numbers used in the text

^b^Patient is reported in (Lines et al 2014)

*Reference range of the laboratory Genetic Metabolic Diseases, AMC, Amsterdam, where all tests were performed except measurements of very long-chain fatty acids and phytanic acid levels which were performed by various metabolic laboratories for the different patients with similar reference values

At the mild end of the clinical spectrum, in the adolescent or young adult period, the distinction between mild ZSDs and AMACR or SCPx deficiency can be difficult to make in patients with cerebellar ataxia, peripheral neuropathy and retinopathy. Because VLCFA levels in mild ZSDs can be normal or even normalize with age (Berendse et al 2015), increased pristanic acid levels can be the only clear abnormality in plasma. In combination with the clinical presentation, a ZSD is unjustly not considered in these patients until fibroblast studies show a mild peroxisome biogenesis defect. For example, spastic paraplegia and mild deafness in a female patient at age 33 years in combination with repeated increased pristanic acid levels in plasma raised the suspicion of AMACR deficiency. Analysis of the AMACR gene, however, did not reveal pathogenic mutations. Fibroblasts were sent in for SCPx enzyme analysis. SCPx activity was within the reference range, but further peroxisomal screening revealed a mild ZSD caused by bi-allelic mutations in *PEX6* (see Table 3, patient 3). In addition, a patient with an acute neurodegenerative disease course and with abnormal VLCFAS all highly suggestive of X-ALD, was diagnosed as a late onset ZSD after peroxisomal studies in fibroblasts (Tran et al 2014).

Fibroblasts studies can also differentiate between the different types of RCDP, which are clinically indistinguishable especially at the severe end of the spectrum and which all have a plasmalogen deficiency in common (Wanders and Waterham 2006a, b; Braverman and Moser 2012; Buchert et al 2014; Baroy et al 2015). In addition, it can classify patients with a Refsum-like phenotype and increased phytanic acid levels in the RCDP spectrum (van den Brink et al 2003) and show that the patient does not suffer from classical Refsum disease but an attenuated form of RCDP type 1 (see Table 3, patient 6). For example, repeatedly very high phytanic acid levels (over 700 μM) were found in plasma of a 9 year old girl with neurological symptoms, suggesting classical Refsum disease. Fibroblast studies did not only reveal a deficient phytanic acid alpha-oxidation, but also a reduced DHAPAT activity and the presence of unprocessed 3-ketoacyl-CoA thiolase on Western blot. Combined these results pointed to a PEX7 defect, which was confirmed by molecular analysis.

Peroxisomal functions in fibroblasts can be abnormal in peroxisomal patients with normal peroxisomal metabolites in blood. Several patients with DBP or ACOX1 deficiency or a ZSD have been reported with normal peroxisomal parameters in blood in whom diagnosis was only established or confirmed by studies in fibroblasts (Soorani-Lunsing et al 2005; Rosewich et al 2006; McMillan et al 2012; Lines et al 2014; Ratbi et al 2015). Especially in milder DBP patients the only identifiable defect in blood and fibroblasts can be DBP activity and expression (see Table 3, patient 5), which is a well-recognized diagnostic pitfall for this disorder. At the very mild end of the ZSD spectrum, in patients presenting with Heimler syndrome, the only identified abnormality in the extensive peroxisomal screening in blood and fibroblasts performed, was the aberrant peroxisomal staining with immunofluorescence microscopy analysis, which exacerbated after culturing the fibroblasts at 40 °C. This means that when the clinical symptoms of a patient are suggestive of a peroxisomal disorder but peroxisomal metabolites in blood and urine are normal, studies in fibroblasts are still warranted. It must be noted that a ZSD with metabolite abnormalities in blood but normal peroxisomal functions in fibroblasts is also possible. For example, a patient with a defect in PEX10 showed normal presence of functional peroxisomes in fibroblasts (Steinberg et al 2009). Hence, a peroxisomal disorder cannot be fully excluded based on normal peroxisomal studies in fibroblasts alone.

The recently identified disorders of peroxisomal fission can be demonstrated only by microscopy in fibroblasts, since peroxisomal parameters in blood and cells are mostly normal in these disorders. Immunofluorescence microscopy analysis in fibroblasts of patients with a defect of PEX11β, DLP/DRP1 and MFF show a clearly aberrant peroxisomal staining characteristic for the underlying defect (Fig. 2). Fibroblasts with a defect of PEX11β, involved in elongation, show enlarged and elongated peroxisomes at 37 °C, while at 40 °C catalase staining becomes cytosolic (Ebberink et al 2012). Fibroblasts with a defect of DLP/DRP1 and MFF (Waterham et al 2007; Shamseldin et al 2012), involved in fission of peroxisomes, show strings of peroxisomes.

When no mutations are identified by genetic analysis, fibroblast studies can still provide the diagnosis, also allowing biochemical prenatal analysis based on these results. For example, in a boy with severe hypotonia, seizures, polymicrogyria, blindness and dysmorphia, DBP deficiency was suspected based on the abnormal VLCFA and bile acid profiles. DHAPAT activity was analysed and found normal. Subsequently, DNA was sent for mutation analysis of the *HSD17B4* gene but no mutations were identified. Further fibroblast studies were recommended and confirmed unambiguously DBP deficiency in the patient (see Table 3, patient 4).

In case NGS is chosen as the diagnostic approach before biochemical studies are performed, biochemical and functional studies often remain essential. *First*, it is very important to confirm that the right diagnosis has been made for proper clinical management of the patient. *Second*, if they do not involve loss-of-function mutations and have not been functionally characterized previously, pathogenicity of the identified DNA variants has to be proven before genetic counseling can be provided to these families. Obviously, this is also imperative before prenatal diagnosis can be offered. If variants are reported as pathogenic without functional studies to actually prove pathogenicity, it may be detrimental in the diagnostic process of other patients and genetic counselling in their families. For example, whole exome sequencing identified a homozygous c.315A > C variant in *PEX1* in two siblings from consanguineous parents with intellectual disability and dysmorphic features. VLCFA and phytanic acid levels were reported to be normal. Fibroblasts studies revealed no abnormalities in any of the peroxisomal parameters, providing no evidence of a peroxisome biogenesis defect as the underlying cause of the clinical symptoms. On the other hand, it is also important to perform complete studies in fibroblasts to prevent the possibility that a diagnosis with pathogenic mutations is unjustly discarded. For example, a homozygous mutation in *PEX13* was recently identified by whole exome sequencing in a boy with normal psychomotor development, who presented at the age of 5 years with ataxia, dysarthria and learning disability. MRI showed hypomyelinations associated with cerebral and vermian atrophy. Fibroblasts studies were performed but revealed completely normal DHAPAT activity, VLCFAs, C26:0 lyso PC and immunoblot profiles for ACOX1 and 3-ketoacy-CoA thiolase (Table 3, patient 2). However, catalase immunofluorescence microscopy analysis revealed a mosaic pattern composed of cells with a normal peroxisomal staining, cells with a reduced number of peroxisomes and cells without import-competent peroxisomes showing that the patient indeed suffered from a ZSD. At 40 °C all catalase staining became cytosolic allowing complementation studies at this temperature and confirmation of the PEX13 defect (Submitted for publication). *Third*, DNA variants, which are unlikely to be pathogenic according to prediction programs, can still be disease-causing, which can be demonstrated by biochemical and functional studies. Based on molecular analysis alone these patients may remain without diagnosis and therefore without treatment. For example, in a 5-year old boy presenting with hypotonia and abnormal pain sense in the legs, secondary enuresis and normal cognition, X-ALD was suspected after VLCFA analysis in blood was abnormal. This suspicion initially seemed not confirmed by mutation analysis of *ABCD1*, which revealed a hemizygous c.1-22C > T variant that had not been reported previously but was not predicted as pathogenic. Fibroblast studies, however, showed unambiguously that the patient suffered from X-ALD since ALDP expression was absent in most but not all cells and VLCFAs were abnormal in repeated analyses (see Table 3, patient 7). *Fourth*, functional studies can provide information about the severity of the defect and can therefore have prognostic value for patients, their parents and their physicians. *Fifth*, if mutations in novel peroxisomal genes are identified, the defect has to be characterized by biochemical studies in blood and functional studies in fibroblasts.

When (one of) the causative mutations cannot be detected by DNA analysis in patients with a ZSD, DBP or ACOX1 deficiency, biochemical prenatal testing is possible, but only after biochemical characterization in fibroblasts of the index patient. In chorionic villous biopsy material, measurement of DBP and ACOX1 activity is possible and in case of a ZSD, DHAPAT activity is measured and immunoblot analysis of ACOX1 and 3-ketoacyl-CoA thiolase performed. In addition, VLCFA analysis and immunofluorescence microscopy analysis is performed in cultured chorionic villous cells. These last two tests can also be performed in cultured amniocytes but this is not preferred because the interpretation is more difficult and only two tests can be performed as opposed to four different tests in chorionic villous material.

## Concluding remarks

Much of our knowledge on peroxisomal functions and the consequences of peroxisomal dysfunction is based on biochemical investigations in tissues, body fluids and fibroblasts of patients with peroxisomal disorders. Biochemical studies followed by functional studies in fibroblasts are crucial in the traditional diagnostic work-up for these disorders, which mostly starts with a clinical suspicion of a peroxisomal disorder. Also when NGS techniques are used, biochemical and functional studies often remain necessary to confirm the diagnosis, prove pathogenicity of previously uncharacterized mutations and to characterize the severity of the defect. Thus, biochemical and functional studies are imperative for proper clinical management of the patients and genetic counseling in the family.
